# Cyclic Deformation of Ultra-Fine Grained Commercial Purity Aluminum Processed by Accumulative Roll-Bonding

**DOI:** 10.3390/ma6083469

**Published:** 2013-08-13

**Authors:** Charles C.F. Kwan, Zhirui Wang

**Affiliations:** 1 Department of Materials Science and Engineering, University of Toronto, 184 College Street, Toronto, Ontario M5S 3E4, Canada; E-Mail: zhirui.wang@utoronto.ca; 2 Department of Mechanical Engineering, Hong Kong University of Science and Technology, Clearwater Bay, Hong Kong, China

**Keywords:** ultrafine grained microstructure, accumulative roll-bonding, cyclic deformation, microstructure stability, shear banding

## Abstract

Accumulative Roll-Bonding (ARB) is one of the more recently developed techniques capable of producing bulk ultra-fine grained (ufg) metals. There are still many aspects of the behavior of ufg metals that lacks an in-depth understanding, such as a generalized view of the factors that govern the cyclic deformation mechanism(s). This study aims to advance the understanding of the cyclic deformation behavior of ufg metals through the systematic investigation of ARB processed aluminum upon cyclic loading. It was found that the cyclic softening response often reported for ufg metals is largely influenced by the microstructure stability as the cyclic softening response is facilitated by grain coarsening which becomes inhibited with highly stable microstructure. On one hand, shear bands resembling braids of dislocations trespassing multiple grains have been observed to operate for the accommodation of the imposed cyclic strain in cases where grain coarsening is largely restricted. On the other hand, it was found that the microstructure stability can be overcome at higher applied cyclic plastic strain levels, leading to grain coarsening and thus a cyclic softening response. The findings in this study have further confirmed that the cyclic softening behavior found in many ufg metals, which may be detrimental in practical applications, can be inhibited by improvements in the microstructure stability.

## 1. Introduction

The enhanced properties of ultra-fine grained (ufg) metals, e.g., improved mechanical strength, have interested many material scientists in the past decades. Frequently, the progress toward the practical applications of ufg metals is impeded by (i) the lack of ability to efficiently produce ufg metals in bulk form without introducing a large amount of defects; and (ii) the lack of deep understanding of the behavior of these metals under various external influences. A number of recently developed techniques, such as the Accumulative Roll Bonding (ARB) process introduced by Saito *et al.* in [1,2], are strong candidates for the production of bulk ufg metals. The ARB process involves repeated roll-bonding of an assemblage of plates in order to introduce severe plastic deformation into the bulk. The detail of the ARB process can be found in [1,2] and will not be repeated here.

On the other hand, there is still a lack of deep understanding of the behavior of ufg metals under various external influences, such as in the case of the cyclic deformation response and behavior. The small grain sizes found in ufg metals have a considerable effect on its response and behavior with respect to their conventional grain sized counterparts, *i.e.*, the small grain size may affect the formation of common dislocation structures associated with deformation. For one, the formation of dislocation cells upon deformation is reportedly hindered in face centered cubic metals with sub-micron grain sizes [3]. Similarly, sub-micron grain sizes restrict the formation of arrays of dislocation walls, which includes various well known mechanisms of cyclic strain accommodation in metals with medium to high stacking fault energy such as persistent slip bands [4]. Consequently, non-conventional cyclic strain accommodation mechanisms are expected to be in operation in the case of ufg metals. Correspondingly, deviation of the cyclic deformation response from that of its conventional counterpart is anticipated. The present study is aimed at advancing the understanding of the cyclic deformation behavior of ufg metals with specific focus on ARBed (*i.e.*, ARB processed) ufg metals. This study will focus on ARBed commercial purity (CP) aluminum. Comparisons will also be drawn with other ARBed ufg metals as well as with ufg metals processed by other techniques in an attempt to provide a generalized view of the cyclic deformation behavior of ufg metals as a whole.

To the best of the authors’ knowledge, this is the first report of its kind on the cyclic deformation response and behavior of ARBed CP aluminum in literature. However, there are a number of reports (e.g., [5,6,7]) on the cyclic deformation behavior of ufg CP aluminum processed with the Equal Channel Angular Pressing (ECAP) technique—a method often considered (e.g., in [8]) to be one of the most developed of the severe plastic deformation techniques. On one hand, grain coarsening and the associated cyclic softening response upon cyclic deformation have been reported for the ECAPed CP aluminum in [5,6]. Höppel *et al.* [6] have further specified that grain coarsening upon cyclic deformation occurs along the plane of maximum shear stress, and consequently they have suggested that this is a form of macroscopic shear banding. Moreover, surface damage due to shear banding was also reported in [6] but its relationship with the macroscopic shear banding is unclear. On the other hand, Wong *et al.* [7] did not observe any noticeable grain coarsening in their ECAPed CP aluminum, although they did observe surface damage relating to shear banding [7]. Instead, Wong *et al.* [7] observed shear bands with microstructure similar to a braid of dislocation that trespass multiple grains. Whether the shear banding reported by Wong *et al.* [7] and Höppel *et al.* [6] represent different stages of the same mechanism or distinct mechanisms is yet to be clear. Despite the difference in the observed micro-mechanism, ECAPed CP aluminum demonstrates varying degrees of cyclic softening after a period of cyclic hardening, a behavior reported commonly in [5,6,7]. The similarities in the cyclic deformation response and behavior between ECAPed and ARBed copper have been shown in a previous work [9]; thus it would be interesting to see not only the cyclic deformation response and behavior of ARBed CP aluminum in the present work but also its comparison with other ARBed metals, e.g., copper, and ECAPed CP aluminum reported in literature.

## 2. Materials and Experiments

The experiment of this study was conducted on plates of CP aluminum (AA1100) after 4, 6, and 8 passes of ARB (labeled 4p, 6p, and 8p respectively) received from the Korean Institute of Materials Science. The composition of the received material in weight percentage is as follows: ~99.1% aluminum, 0.17% silicon, 0.57% iron, 0.11% copper, 0.001% magnesium, and 0.02% titanium. The microstructure was examined with the Electron Channeling Contrast Imaging (ECCI) technique under an analytical SEM and with conventional transmission electron microscope (TEM) technique. TEM thin foils were prepared by twin jet electro-polishing with an electrolyte consisting of 30% HNO_3_ and 70% methanol. Samples intended for ECCI observation were prepared with standard metallographic method followed by electro-polishing using the electrolyte employed in twin jet electro-polishing above.

Tension-Tension cyclic deformation tests were carried out with dog-bone samples machined according to 50% of the ASTM B557M subset design. Cyclic deformation tests were carried out under both load controlled and total strain controlled schemes utilizing a sinusoidal waveform. Load controlled tests were carried out at a frequency of 1 Hz with peak stress equal to 90%, 100%, and 110% of the samples’ respective yield strength with an stress ratio (R) of 0.05. Total strain controlled tests were carried out at a frequency of 0.5 Hz with total strain amplitudes ranging from 1.7 × 10^−3^ to 4.1× 10^−3^.

## 3. Results and Discussion

### 3.1. Initial Microstructure

**Figure 1:**
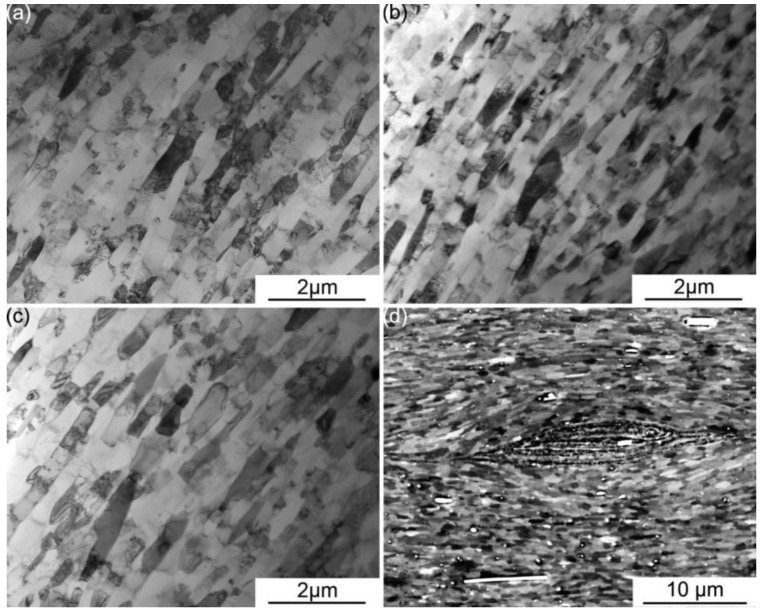
Microstructure of the (**a**) 4p; (**b**) 6p; and (**c**) 8p Accumulative Roll Bonding (ARB) CP aluminum samples as viewed under bright field transmission electron microscope (TEM) imaging; (**d**) an Electron Channeling Contrast Imaging (ECCI) micrograph of an 8p samples showing the composite nature of the present microstructure.

The microstructures of the ufg 4p, 6p, and 8p aluminum samples are consistent with that reported previously in literature, e.g., [10,11,12,13]. The grain geometry as viewed from the transverse view under a TEM, shown in Figure 1a–c respectively, consists of ultra-fine pancake shaped grains. The relatively low dislocation density within these pancake shaped grains suggests the occurrence of a dynamic recovery process during ARB processing; a notion similarly suggested by Li *et al.* [13] in the case of ARBed aluminum. More importantly, the as-ARBed CP aluminum exhibits a composite natured microstructure with three constituents of varying grain sizes, a concept that has been previously demonstrated for ARBed copper [14]. The three constituents in the as-ARBed microstructure are: (i) the ufg matrix as seen in Figure 1a–c in which grain size are similar to those reported in [10,11,12]; (ii) the primary discontinuities—pockets along the interlayer surface consisting of heavily deformed materials left behind due to the roll-bonding mechanism; and (iii) the pre-existing coarse grains, *i.e.*, grains that have selectively coarsened around the primary discontinuities due to the severe plastic deformation and heat involved during ARB processing [14]. The clear distinction of these three constituents within the as-ARBed CP aluminum microstructure is clearly shown using the ECCI technique in Figure 1d. It should be noted that intermetallic particles consisting of aluminum, iron, and silicon found in the received CP aluminum appear as bright (white) spots in Figure 1d due to the use of the backscattered electron detector for the ECCI technique. More interestingly, the pre-existing coarse grains observed here have much smaller dimensions compared to those revealed in the ARBed copper in [14]. However, there is still a noticeable difference between the grain dimensions of these pre-existing coarse grains and that of the matrix grains in the current material. The lowered extent of grain coarsening during the ARB process is likely a result of the higher microstructure stability, from both the dynamic recovery nature of aluminum [13] and the alloying chemistry, compared to the case of ARBed OFHC copper in [14].

### 3.2. Cyclic Deformation Response

**Figure 2:**
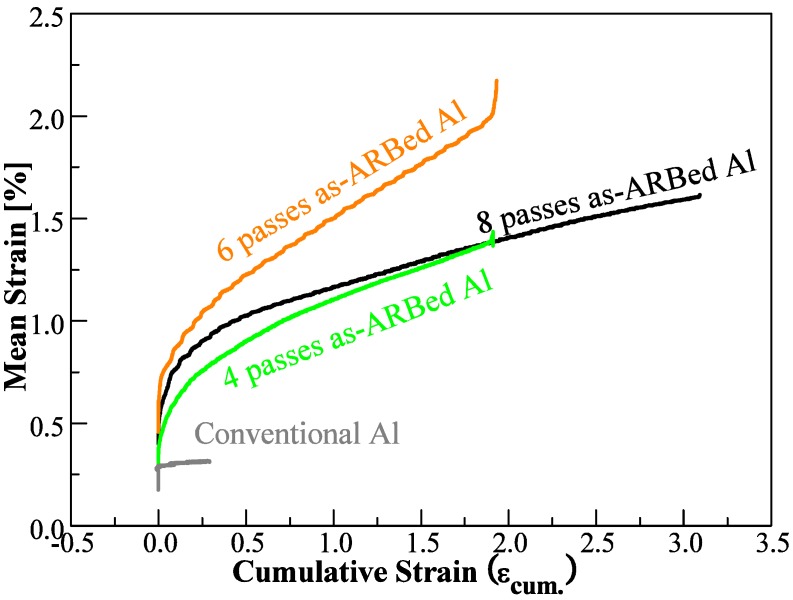
Plot of the mean strain of each cycle demonstrating the cyclic creep behavior of 4p, 6p, and 8p ARBed CP aluminum samples under load controlled testing with σ_peak_ = 100% of the respective yield strength. The curve for conventional aluminum is also shown for comparison purposes.

All of the ufg ARBed CP aluminum studied demonstrated significant cyclic creep response under load controlled and similar stress relaxation response under total strain controlled tension-tension cyclic testing. The cyclic creep response is apparent when plotting the mean strain *versus* the number of cycles of loading, as shown in Figure 2 for a few representative samples. It should be noted that cyclic creep response was not observed in the case of the conventional grain sized CP aluminum samples, also shown in Figure 2 for comparison purposes. A point to note is that the plastic strain accommodated in each cycle is independent of the mean strain trend shown in Figure 2. In essence, the trend in Figure 2 simply shows the shift of the entire hysteresis loop toward a more positive mean strain. More interestingly, the cyclic creep response of the 8p samples is noticeably less prominent in terms of both the extent of the cyclic creep response and the rate of the cyclic creep process in comparison with the response of 4p and 6p samples.

**Figure 3:**
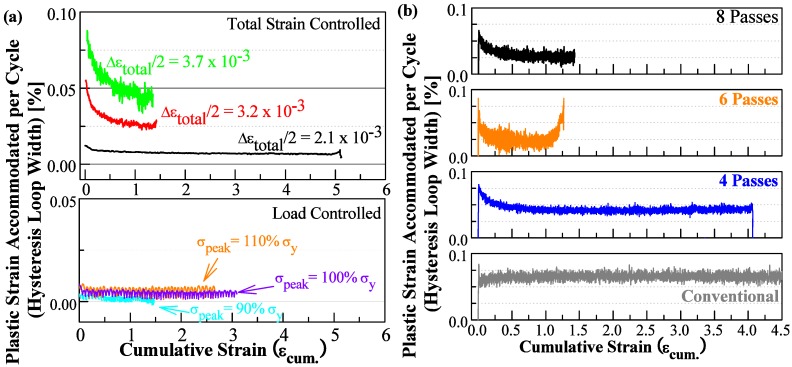
Plots of the trend of hysteresis loop width representing the cyclic hardening/softening behavior of ARBed CP aluminum of (**a**) an 8p sample upon cyclic deformation with different parameters; and (**b**) of 4p, 6p, and 8p samples upon cyclic loaded under total strain controlled with an amplitude of Δε_total_/2 = 3.2 × 10^−3^.

The cyclic hardening/softening response under both load controlled and total strain controlled tests were assessed using the trends of plastic strain accommodated in each cycle, *i.e.*, the hysteresis loop width. Plots showing such trends for a number of representative samples under different processing and loading conditions are shown in Figure 3. ARBed CP aluminum samples show little to no cyclic hardening/softening behavior when tested under load control, as shown in Figure 3a for representative 8p samples. Upon closer inspection, the lack of any cyclic hardening/softening response is most likely the result of the nearly zero extent of plastic strain involved in each cycle during these tests. The level of plastic strain accommodation in each cycle is raised significantly with the employment of the total strain controlled tests. The ARBed CP aluminum samples tested under total strain controlled tests all demonstrated various extent of cyclic hardening response early in the cyclic lifespan, as shown in Figure 3a for 8p samples upon cyclic loading with various cyclic loading parameters and in Figure 3b for the 4p, 6p, and 8p samples tested at a total strain amplitude Δε_total_/2 = 3.2 × 10^−3^. However, the cyclic deformation response following this initial period of cyclic hardening is different for the 6p samples compared to that of the 4p and 8p samples. A cyclic saturation response follows the initial cyclic hardening response in the case of the 4p and 8p samples, whereas a cyclic softening response follows the initial period of cyclic hardening in the case of the 6p samples, see Figure 3b. Considering the cyclic softening response initiated in the late stage of the cyclic lifespan for the 6p samples, it is likely that such cyclic softening response is due to the occurrence of macro-scale plastic instability within the gauge length.

### 3.3. The Activated Micro-Mechanisms

**Figure 4:**
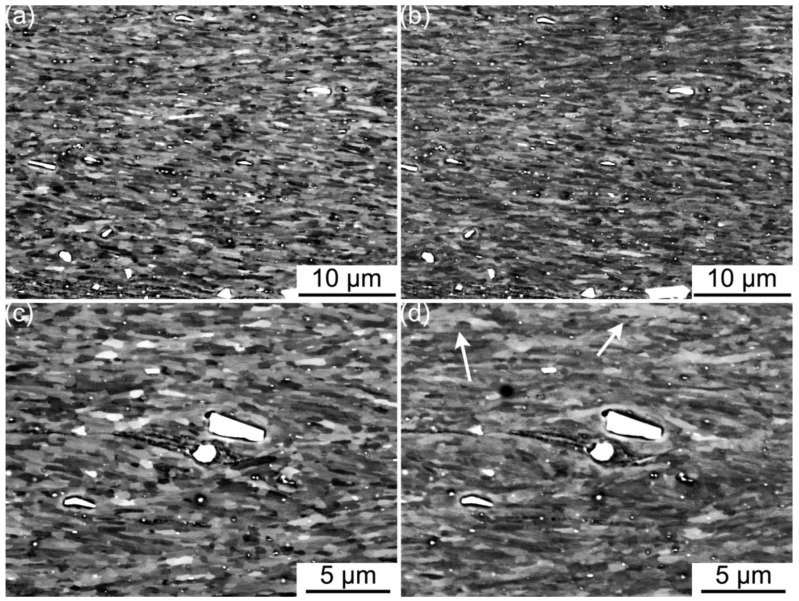
ECCI micrograph showing an 8p ARBed CP aluminum sample upon cyclic loaded under total strain controlled with an amplitude of Δε_total_/2 = 3.7 × 10^−3^, (**a**) prior to any cyclic loading; and (**b**) after cyclic fracture at 1333 cycles at the same location as (**a**). (**c**,**d**) is a similar set at higher magnification showing the minor coarsening observed (arrowed in (**d**)).

The extent of cyclic softening has been previously linked to the extent of grain coarsening in ufg metals [15]. The lack of a significant cyclic softening response in the current case of ARBed CP aluminum suggests that grain coarsening is unlikely to have occurred in this material. Indeed, large scale grain coarsening was not detected upon cyclic loading in the ARBed CP aluminum studied here, as shown in Figure 4. However, some minor scale grain coarsening can be seen especially within those samples cyclically deformed at the higher strain amplitudes, e.g., arrowed in Figure 4d. Clearly, grain coarsening is possible in ARBed CP aluminum upon cyclic deformation and it has not been fully inhibited by the relatively high microstructure stability of this material. Such a notion that grain coarsening is indeed possible is further confirmed with the formation of the pre-existing coarse grain constituent during ARB processing. Both cases listed above clearly show that the potential for grain coarsening upon mechanical stimulation is possible and is actually present in the current ARBed CP aluminum samples. However, the dynamically recovered microstructure and more importantly the additional alloying elements have largely inhibited the grain coarsening process, as demonstrated in the experimental results of the present work.

The lack of significant grain coarsening observed in this study is contrary to the case of ECAPed CP aluminum reported by Höppel *et al.* in [5,6]. However, the phenomenon observed is similar to that reported in [16], in which the extent of cyclic softening decreases with increasing microstructure stability via the increase of magnesium content in ECAPed Al-Mg alloy. On the other hand, the current observations suggest that increasing mechanical stimulation can aid in overcoming the higher microstructure stability. More specifically, the trend observed in this study suggests that significant grain coarsening can occur with a sufficiently high imposed cyclic plastic strain per cycle. On one hand, even at the highest strain level tested in this study, *i.e.*, Δε_total_/2 = 4.1 × 10^−3^, the imposed cyclic plastic strain, starting at Δε_plastic._/2 = 5 × 10^−4^ and decreases with increasing cyclic life in this case, is not enough for large scale grain coarsening to occur in the current material. However, the significant grain coarsening reported for ECAPed aluminum in [6] in which the imposed cyclic plastic strain Δε_plastic._/2 = 1 × 10^−3^, is at least doubled the cyclic plastic strain in the current study, further accentuates the effect of the imposed cyclic plastic strain on overcoming the microstructure stability.

The re-activation of shear bands formed during ARB processing has been reported as one of the active mechanisms upon cyclic deformation of ARBed coppers [14]. The shear bands formed during ARB processing are similarly present in the current ARBed aluminum and can be seen in Figure 4. Unlike the case of ARBed copper in [14], there is no apparent surface damage associated with shearing banding even after cyclic deformation to fracture in the current ARBed aluminum. However, it should be noted that the formation of surface damages may be constrained by the presence of an oxide layer. That is to say, the re-activation of the shear bands formed during ARB processing cannot be excluded as one of the active mechanisms upon cyclic deformation simply based on the lack of apparent surface damage. Furthermore, the re-activation of the shear bands from ARB processing is thought to provide stress concentrations which aid in promoting grain coarsening upon cyclic loading [17]. As such, it is very likely that the shear bands from ARB processing have indeed re-activated to an extent allowing for the minor grain coarsening observed in the current material.

**Figure 5:**
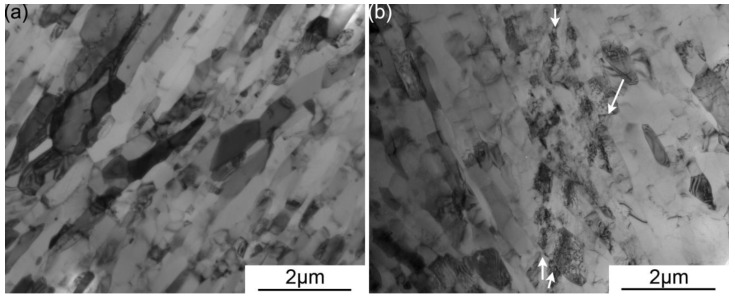
Bright field TEM micrographs of 8p ARBed CP aluminum samples (**a**) in the as-ARBed form; and (**b**) after cyclic loading under load controlled with peak stress σ_peak_ = 90% σ_y_ and R = 0.05 after cyclic fracture at 44404 cycles. The observed shear bands in the form of braids of dislocations are arrowed in (**b**).

On a smaller length scale, the ultra-fine grain sizes have indeed suppressed the formation of arrays of dislocation walls in the ARBed CP aluminum, as shown in Figure 5 for a representative sample. This observation is in good agreement to the findings of Glazov and Laird in [3]. The current submicron grain size is not expected to be small enough to fully inhibit dislocation activities. However, the dislocation density within the grains upon cyclic deformation has remained similar to that of the as-ARBed microstructure. The seemingly unchanged dislocation density may be the result of a dynamic recovery process upon cyclic deformation, a case similar to that observed as a result of ARB processing.

Interestingly, shear bands similar to those reported by Wong *et al.* in [7] have been observed in several cases in this study, e.g., Figure 5b. The observation of such braids of dislocations trespassing multiple grains was more prevalent in the load controlled samples, *i.e.*, those that have experience a lower cyclic plastic strain. It should be emphasized that the occurrence of this type of shear bands are limited in quantity and are found only after intensive search under TEM observation; a case similar to that reported by Wong *et al.* [7]. Shear bands in this form and grain coarsening upon cyclic loading have yet to be observed within the same sample of severe plastic deformed ufg metals. The cases of ECAPed aluminum reported in [5,6,7] and the instances of ECAPed and ARBed high purity copper reported in [6,12,15,17,18,19,20] have reported either shear banding or grain coarsening but not both. With consideration of the above, shear banding observed in this case seem to only activate to accommodate the imposed cyclic deformation when favorable conditions are absent for both grain coarsening and conventional dislocation mechanisms. That is to say, shear banding seems to be favorable in ufg materials with high microstructure stability in which the small grain sizes restrict conventional dislocation mechanisms and the high microstructure stability inhibits grain coarsening. The high stacking fault energy of aluminum may have also allowed for the large amount of cross-slip necessary to form a relatively straight braid of dislocations through multiple grains of varying orientation. The lack of observation of similar shear bands in ECAPed technical purity copper in literature, e.g., [21,22,23], whereby large scale grain coarsening were also absent is likely a result of the lower cross-slip potential of copper. It is arguable that the activation of the “braid” like shear bands are related to the asymmetrical control spectra since a lack of plastic strain accommodation were recorded for those samples (e.g., Figure 3a) yet a noticeable cyclic creep or stress relaxation response was recorded (e.g., Figure 2). However, this possibility seems unlikely based on the evidence in literature. For one, Wong *et al.* [7] observed similar shear bands under symmetrical cyclic schemes. Moreover, this type of shear bands has not been observed in ARBed high purity copper in tension-tension cyclic testing in previous unpublished work by the present authors where cyclic creep response was present as well.

### 3.4. Correlating the Micro-Mechanism and the Cyclic Deformation Response

The ultra-fine grain sizes have indeed suppressed the formation of arrays of dislocation walls in the ARBed CP aluminum, but the grain size in the current case is not expected to be small enough to fully inhibit dislocation activities. As such, the initial period of cyclic hardening observed in the current ARBed aluminum sample is very likely due to dislocation activities and interactions with other dislocations or grain boundaries. The cyclic saturation that follows is likely the result of dynamic recovery within the microstructure as the active dislocation mechanism becomes increasingly exhausted. It should be noted that the composite nature of the ARBed microstructure leads to a heterogeneous stress distribution in the volume of the gauge length upon mechanical loading. This heterogeneity allows for different mechanisms to be active simultaneously at different locations along the gauge length. As such, the cyclic saturation is likely a result of simultaneous operation of dislocation interactions and dynamic recovery at different locations along the gauge length. However, it is probable that the occurrence of minor grain coarsening at different locations may also have a role in the cyclic saturation response. Grain coarsening is known to lead to a cyclic softening response, as shown in [14,15]. As grain coarsening is known to occur at location of stress concentration and hence strain localization, the accommodation of a notable portion of the imposed cyclic strain by these coarsened grains is likely and may produce a perceptible effect on the total cyclic deformation response although it is a localized event. Furthermore, although the extent of grain coarsening is relatively minor in the current case, the population of coarsened grains may still be sizable when taking the entire gauge length volume into consideration leading to a noticeable effect on the response. In the current case, such minor grain coarsening may have partial contribution, in combination with dynamic recovery, in balancing the cyclic hardening response from dislocation interactions elsewhere.

The peculiar cyclic deformation response of 6p ARBed aluminum sample is of even more interest. Cyclic failure in the 4p and 8p samples is thought to occur as the capability to accommodate strain is reduced due to increasing dislocation interactions within the small grains and the lack of significant grain coarsening to relieve such situation. Furthermore, the fracture process occurred abruptly, often within a small number of cycles in the 4p and 8p samples. Contrarily, both of the 6p samples tested consistently exhibit a significant cyclic softening response in the late stage cyclic lifespan prior to complete fracture. Such late stage softening response is undoubtedly related to the early development of a fatal crack. More interestingly, the process from the development of this fatal crack to final fracture in the 6p samples consumes a larger proportion of the cyclic lifespan than the cases of the 4p and 8p samples. Although further confirmation is needed, it is likely that grain coarsening occurred in massive numbers after the onset of plastic instability, as a result of the development of a fatal crack, and hence leading to an increase in ductility and a longer crack development process. Interestingly, the microstructure of 6p CP aluminum samples have been previously reported to be less stable than that of 8p samples despite the similar grain size [12]. As such, the driving force for grain coarsening is expected to be even higher in the case of the 6p samples with respect to the 4p and 8p samples. Furthermore, the onset of plastic instability not only provides a higher stress/strain level but also creates a state of stress concentration/strain localization; both the former and the latter have been shown to further promote grain coarsening upon cyclic loading in ARBed metals [14,17].

It is doubtful that a similar argument to the above can be used to explain the peculiar cyclic creep response when comparing 6p and 8p samples. Although the extent of cyclic creep response seems to vary with the microstructure stability of the as-ARBed material as reported in [12], it is unlikely to be a direct consequence. For one, it is shown in the present work that the cyclic creep mechanism is not related to any apparent microstructure change, such as the grain coarsening that is related to the cyclic softening behavior. Moreover, the present investigation shows that the 4p samples also demonstrated a higher rate of the cyclic creep process in comparison to 8p samples. However, the former have accumulated significantly lesser strain compared to the latter, and hence should have similar (if not higher) microstructure stability compare to that of the latter, a notion shown to be accurate in [12]. Thus, it seems more fitting that the trend in cyclic creep is dependent on the amount of mobile dislocations within the grains for dislocation interactions that eventually leads to irreversible dislocation structure evolution manifesting in the form of a cyclic creep response. Since a dynamic recovery process is known to occur during the ARB processing between the 6th and 8th pass [12], the 8p sample would likely have fewer mobile dislocations compared to the cases of 4p and 6p samples, hence leading to a much less prominent cyclic creep response.

## 4. Conclusions

Although the cyclic deformation response of various ARBed materials has been reported from time to time, the present study has provided a first look of the cyclic deformation behavior of ufg CP aluminum produced by the ARB process. The ARBed CP aluminum studied here has demonstrated many traits that have extended the understanding of the cyclic deformation behavior of ufg metals as a whole. In contrast to the case of ARBed copper and other ECAPed metals reported in literatures, the ARBed CP aluminum show a lack of significant grain coarsening upon cyclic deformation and hence the lack of a cyclic softening response. However, the minor grain coarsening observed at higher imposed cyclic plastic strain provides confirmation that grain coarsening is possible but simply suppressed; most likely by the relatively stable microstructure of the current material. On the other hand, shear bands resembling braids of dislocations trespassing multiple grains are apparent at lower cyclic plastic strain in the current material. It is therefore rational to conclude that this form of shear banding is only activated in cases where the imposed cyclic plastic strain is too low to overcome the microstructure stability for the activation of grain coarsening upon cyclic deformation.

The various cyclic strain accommodation mechanisms observed here further highlight the importance of microstructure stability on the cyclic deformation response of ARBed metals. Furthermore, consolidation of the current observations and those in literature would suggest that the dependence of cyclic deformation behavior, more specifically the extent of the cyclic softening response, on the microstructure stability is likely applicable to other submicron grain sized metals processed by other techniques.

## References

[1] Saito Y., Tsuji N., Utsunomiya H., Sakai T., Hong R.G. (1998). Ultra-fine grained bulk aluminum produced by accumulative roll-bonding (ARB) process. Scr. Mater..

[2] Saito Y., Utsunomiya H., Tsuji N., Sakai T. (1999). Novel ultra-high straining process for bulk materials—Development of the accumulative roll-bonding (ARB) process. Acta Mater..

[3] Conrad N., Jung K. (2006). Effects of grain size from millimeters to nanometers on the flow stress of metals and compounds. J. Electron. Mater..

[4] Glazov M.V., Laird C. (1995). Size effects of dislocation patterning in fatigued metals. Acta Metall. Mater..

[5] May J., Amberger D., Dinkel M., Höppel H.W., Göken M. (2008). Monotonic and cyclic deformation behavior of ultrafine-grained aluminium. Mater. Sci. Eng. A.

[6] Höppel H.W., Xu C., Kautz M., Barta-Schreiber N., Langdon T.G., Mughrabi H., Zehetbauer M.J., Valiev R.Z. (2004). Cyclic Deformation Behaviour and Possibilities for Enhancing the Fatigue Properties of Ultrafine-Grained Metals. Proceedings of Second International Conference on Nanomaterials by Severe Plastic Deformation.

[7] Wong M.K., Kao W.P., Lui J.T., Chang C.P., Kao P.W. (2007). Cyclic deformation of ultrafine-grained aluminum. Acta Mater..

[8] Valiev R.Z., Estrin Y., Horita Z., Langdon T.G., Zehetbauer M.J., Zhu Y.T. (2006). Producing bulk ultrafine-grained materials by severe plastic deformation. J. Manag..

[9] Kwan C.C.F., Wang Z. (2011). On the Cyclic Deformation Response and Microstructural Mechanisms of ECAP and ARB Copper—An Overview. Mater. Sci. Forum.

[10] Huang X., Tsuji N., Hansen N., Minamino Y. (2003). Microstructural evolution during accumulative roll-bonding of commercial purity aluminum. Mater. Sci. Eng. A.

[11] Kim Y.S., Lee T.O., Shin D.H. (2004). Microstructural Evolution and Mechanical Properties of Ultrafine Grained Commercially Pure 1100 Aluminum Alloy Processed by Accumulative Roll-Bonding (ARB). Mater. Sci. Forum.

[12] Kwan C., Wang Z., Kang S.B. (2008). Mechanical behavior and microstructural evolution upon annealing of the accumulative roll-bonding (ARB) processed Al alloy 1100. Mater. Sci. Eng. A.

[13] Li B.L., Tsuji N., Kamikawa N. (2006). Microstructure homogeneity in various metallic materials heavily deformed by accumulative roll-bonding. Mater. Sci. Eng. A.

[14] Kwan C.C.F., Wang Z. (2011). A Composite Nature of Cyclic Strain Accommodation Mechanisms of Accumulative Roll Bonding (ARB) Processed Cu Sheet Materials. Mater. Sci. Eng. A.

[15] Höppel H.W., Zhou Z.M., Mughrabi H., Valiev R.Z. (2002). Microstructural study of the parameters governing coarsening and cyclic softening in fatigued ultrafine-grained copper. Philos. Mag. A.

[16] Höppel H.W., May J., Göken M. (2008). Cyclic Deformation Behaviour and Fatigue Lives of Ultrafine-Grained Aluminium-Magnesium Alloys. Mater. Sci. Forum.

[17] Kwan C.C.F., Wang Z. (2013). Strain Incompatibility and its influence on the cyclic deformation behaviour of ARB Copper. Philos. Mag..

[18] Furukawa Y., Fujii T., Onaka S., Kato M. (2009). Cyclic Deformation Behavior of Ultra-Fine Grained Copper Produced by Equal Channel Angular Pressing. Mater. Trans..

[19] Maier H.J., Gabor P., Gupta N., Karaman I., Haouaoui M. (2006). Cyclic stress–strain response of ultrafine grained copper. Int. J. Fatigue.

[20] Li X.-W., Jiang Q.-W., Ying W., Wang Y., Umakoshi Y. (2008). Stress-Amplitude-Dependent Deformation Characteristics and Microstructures of Cyclically Stressed Ultrafine-Grained Copper. Adv. Eng. Mater..

[21] Lukáš P., Kunz L., Svoboda M. (2007). Effect of Low Temperature on Fatigue Life and Cyclic Stress-Strain Response of Ultrafine-Grained Copper. Metall. Mater. Trans. A.

[22] Kunz L., Lukáš P., Svoboda M. (2006). Fatigue strength, microstructural stability and strain localization in ultrafine-grained copper. Mater. Sci. Eng. A.

[23] Xu C.Z., Wang Q.J., Zheng M.S., Li J.D., Huang M.Q., Jia Q.M., Zhu J.W., Kunz L., Buksa M. (2008). Fatigue behavior and damage characteristic of ultra-fine grain low-purity copper processed by equal-channel angular pressing (ECAP). Mater. Sci. Eng. A.

